# 重组抗CD25人源化单克隆抗体挽救性治疗糖皮质激素耐药型急性移植物抗宿主病64例疗效分析

**DOI:** 10.3760/cma.j.issn.0253-2727.2023.09.009

**Published:** 2023-09

**Authors:** 雅雪 吴, 德沛 吴, 骁 马, 珊珊 姜, 梦佳 侯, 雨童 景, 彬 刘, 茜 李, 杏 王, 源兵 吴, 晓慧 胡

**Affiliations:** 1 苏州弘慈血液病医院，苏州 215000 Soochow Hopes Hematology Hospital, Suzhou 215006, China; 2 苏州大学附属第一医院，江苏省血液研究所，国家血液系统疾病临床医学研究中心，国家卫生健康委员会血栓与止血重点实验室，苏州 215006 Jiangsu Institute of Hematology, National Clinical Research Center for Hematology Disease, NHC Key Laboratory of Thrombosis and Hemostasis, The First Affiliated Hospital of Soochow University, Suzhou 215006, China

**Keywords:** 造血干细胞移植, 急性移植物抗宿主病, 重组抗CD25人源化单克隆抗体, Hematopoietic stem cell transplantation, Acute graft-versus-host disease, Humanized anti-CD 25 monoclonal antibody

## Abstract

**目的:**

分析重组抗CD25人源化单克隆抗体（人源化CD25单抗）治疗异基因造血干细胞移植后糖皮质激素耐药型急性移植物抗宿主病（SR-aGVHD）的疗效。

**方法:**

纳入2019年6月至2020年10月在苏州弘慈血液病医院接受人源化CD25单抗治疗的64例异基因造血干细胞移植后SR-aGVHD患者，所有患者予人源化CD25单抗1 mg·kg^−1^·d^−1^第1、3、8天各1次，此后根据病情每周1次。在人源化CD25单抗治疗后第7、14、28天进行疗效评估。

**结果:**

64例患者中男38例（59.4％），女26例（40.6％），中位年龄31（15～63）岁。64例SR-aGVHD患者人源化CD25单抗治疗后第7、14、28天有效率分别为48.4％（31/64）、53.1％（34/64）、79.7％（51/64）。肝脏受累是人源化CD25单抗治疗SR-aGVHD第28天疗效不佳的独立危险因素（*OR*＝9.588，95％*CI* 0.004～0.291，*P*＝0.002）。从人源化CD25单抗治疗开始随访，所有患者的中位随访时间为17.1（0.2～50.8）个月。治疗后12、24个月总生存率分别为63.2％（95％*CI* 57.1％～69.3％）、52.6％（95％*CI* 46.1％～59.1％），无病生存率分别为58.4％（95％*CI* 52.1％～64.7％）、49.8％（95％*CI* 43.4％～56.2％），非复发死亡率分别为28.8％（95％*CI* 23.1％～34.5％）、32.9％（95％*CI* 26.8％～39.0％）。多因素分析结果显示，肝脏受累（*OR*＝0.308，95％*CI* 0.108～0.876，*P*＝0.027）和Ⅲ/Ⅵ度aGVHD（*OR*＝9.438，95％*CI* 1.211～73.577，*P*＝0.032）是影响移植后总生存的独立危险因素。

**结论:**

人源化CD25单抗对于异基因造血干细胞移植后SR-aGVHD有较好的疗效。

## Abstract (en)

**Objective:**

To investigate the efficacy of humanized anti-CD25 monoclonal antibody for steroid-refractory acute graft-versus-host disease（SR-aGVHD）in allogeneic hematopoietic stem cell transplantation（allo-HSCT）recipients.

**Methods:**

A total of 64 patients with SR-aGVHD between June 2019 and October 2020 in Suchow Hopes Hematology Hospital were enrolled in this study. Humanized anti-CD25 monoclonal antibodies 1 mg·kg^−1^·d^−1^ were administered on days 1, 3, and 8, and then once per week according to the disease progression. Efficacy was assessed at days 7, 14, and 28 after humanized anti-CD 25 treatment.

**Results:**

Of the 64 patients with a median age of 31（15–63）years, 38（59.4％）were male and 26（40.6％）were female. The overall response（OR）rate of the humanized CD25 monoclonal antibody in 64 patients with SR-aGVHD on days 7, 14, and 28 were 48.4％（31/64）, 53.1％（34/64）, and 79.7％（51/64）, respectively. Liver involvement is an independent risk factor for poor efficacy of humanized CD25 monoclonal antibody for SR-aGVHD at day 28（*OR*＝9.588, 95％ *CI* 0.004–0.291, *P*＝0.002）. The median follow-up time for all patients was 17.1（0.2–50.8）months from the start of humanized CD25 monoclonal antibody therapy. The 1- and 2-year OS rates were 63.2％（95％ *CI* 57.1％–69.3％）and 52.6％（95％ *CI* 46.1％–59.1％）, respectively. The 1- and 2-year DFS rates were 58.4％（95％ *CI* 52.1％–64.7％）and 49.8％（95％ *CI* 43.4％–56.2％）, respectively. The 1- and 2-year NRM rates were 28.8％（95％ *CI* 23.1％–34.5％）and 32.9％（95％ *CI* 26.8％–39.0％）, respectively. The results of the multifactorial analysis showed that liver involvement（*OR*＝0.308, 95％ *CI* 0.108–0.876, *P*＝0.027）and GVHD grade Ⅲ/Ⅳ（*OR*＝9.438, 95％ *CI* 1.211–73.577, *P*＝0.032）were independent risk factors for OS.

**Conclusion:**

Humanized CD25 monoclonal antibody has good efficacy and safety for SR-aGVHD. This study shows that SR-aGVHD with pretreatment grade Ⅲ/Ⅳ GVHD and GVHD involving the liver has poor efficacy and prognosis and requires early intervention.

异基因造血干细胞移植（allo-HSCT）是目前治疗人类多种血液疾病最有效的方法之一，急性移植物抗宿主病（aGVHD）是allo-HSCT的主要并发症和死亡原因[1]–[2]。糖皮质激素是aGVHD的首选治疗方案，但有效率只有约50％[3]–[5]，aGVHD的二线治疗尚无标准方案。抗CD25单克隆抗体可以与IL-2受体α特异性结合而阻断T淋巴细胞激活，从而发挥抗GVHD效应[6]。本研究对苏州弘慈血液病医院2019年6月至2020年10月应用重组抗CD25人源化单克隆抗体（人源化CD25单抗）治疗的64例糖皮质激素耐药型aGVHD（SR-aGVHD）患者进行回顾性分析，初步评估人源化CD25单抗治疗allo-HSCT后SR-aGVHD的疗效。

## 病例与方法

一、研究对象

本研究纳入符合以下条件的患者：①2019年6月至2020年10月在苏州弘慈血液病医院住院的allo-HSCT患者，合并皮肤、肝脏或肠道aGVHD；②在环孢素A（CsA）、他克莫司或霉酚酸酯（MMF）基础上加用甲泼尼龙1～2 mg·kg^−1^·d^−1^一线治疗后出现的SR-aGVHD患者；③以人源化CD25单抗作为二线治疗首选方案。

二、预处理

同胞全相合移植采用改良BuCy（白消安+环磷酰胺）方案；无关供者移植及单倍体移植采用改良BuCy+抗胸腺细胞球蛋白（ATG）方案；骨髓外侵犯患者采用全身放射治疗（TBI）联合Cy方案。

三、移植模式

无关供者移植3例（4.7％），同胞全相合移植12例（18.8％），单倍体移植49例（76.6％）。

四、aGVHD的预防

同胞HLA全相合移植患者采用CsA+短程甲氨蝶呤（MTX）方案预防GVHD，无关供者移植和单倍体移植患者加用MMF[7]。

五、治疗方法

参照2014年美国国立卫生研究院（NIH）专家共识[8]对aGVHD进行诊断和分级。首先应用甲泼尼龙1～2 mg·kg^−1^·d^−1^一线治疗，若3 d内aGVHD进展或5～7 d内评估aGVHD未改善，则诊断为SR-aGVHD，逐渐减停甲泼尼龙并给予人源化CD25单抗进行挽救性治疗（1 mg·kg^−1^·d^−1^，第1、3、8天各1次，此后每周1次，疗程根据病情决定）。

六、疗效判断标准及定义

完全缓解（CR）：所有累及脏器的aGVHD表现完全消失；部分缓解（PR）：至少一个初始受累器官的aGVHD改善（至少降低一个级别）但未达到CR，无其他任何靶器官aGVHD恶化；无效（NR）：任何器官的aGVHD严重程度无改善或至少1个靶器官的aGVHD加重（至少增加1个级别）。CR+PR定义为“有效”。人源化CD25单抗治疗起效时间：从应用人源化CD25单抗治疗开始到aGVHD获得PR的时间。

七、随访

随访资料来自门诊/住院病历及电话随访。末次随访日期为2022年12月5日。总生存（OS）和无病生存（DFS）时间均从人源化CD25单抗治疗开始计算。非复发死亡（NRM）：人源化CD25单抗治疗开始后因复发以外的任何原因导致死亡或失访。

八、统计学处理

使用SPSS 20软件进行统计学分析。分类资料使用卡方检验或Fisher确切概率法，单因素分析*P*<0.05的变量纳入多因素分析，多因素预测模型采用Logistic回归分析，*P*<0.05为差异有统计学意义。生存分析采用Kaplan-Meier法，组间比较单因素分析采用Log-rank检验，*P*<0.05的变量纳入多因素分析，多因素分析采用Cox比例风险回归模型，*P*<0.05为差异有统计学意义。使用GraphPad Prism 9软件绘图。

## 结果

一、患者特征

本研究共纳入64例患者，其中男38例（59.4％），女26例（40.6％）。中位年龄31（15～63）岁。重型再生障碍性贫血6例，原发性骨髓纤维化1例，噬血细胞综合征1例，NK/T细胞淋巴瘤1例，慢性粒-单核细胞白血病1例，骨髓增生异常综合征13例，急性白血病41例，其中慢粒急变T系伴髓系表达1例，急性髓系白血病21例，急性淋巴细胞白血病17例，急性混合细胞白血病2例。中位回输CD34^+^细胞数为3.94（1.25～9.58）×10^8^/kg，CD3^+^细胞数为1.4（0.3～4.3）×10^8^/kg。64例患者基线临床资料见表1。

**表1:** 64例接受重组抗CD25人源化单克隆抗体挽救性治疗SR-aGVHD患者的临床资料

临床特征	结果
年龄［岁，*M*（范围）］	31（15~63）
性别［例（%）］	
男	38（59.4）
女	26（40.6）
诊断［例（%）］	
AML	21（32.8）
ALL	18（28.1）
MAL	2（3.1）
MDS	13（20.3）
SAA	6（9.4）
其他	4（6.3）
造血干细胞来源［例（%）］	
外周血	34（53.1）
骨髓	30（46.9）
供者类型［例（%）］	
同胞全相合	12（18.8）
单倍体	49（76.6）
无关供者	3（4.7）
供者性别［例（%）］	
男	47（73.4）
女	17（26.6）
供患者体血型［例（%）］	
主侧不合	9（14.1）
次侧不合	13（20.3）
主次侧均不合	2（3.1）
血型相合	40（62.5）
受累器官［例（%）］	
单纯皮肤	2（3.1）
单纯肠道	16（25.0）
单纯肝脏	1（1.6）
皮肤+肠道	19（29.7）
皮肤+肝脏	3（4.7）
肠道+肝脏	11（17.2）
皮肤+肠道+肝脏	12（18.8）
受累器官数［例（%）］	
1个	19（29.7）
2个	33（52.6）
3个	12（18.8）
aGVHD分级［例（%）］	
Ⅱ度	13（20.3）
Ⅲ/Ⅵ度	51（79.7）

**注** AML：急性髓系白血病；ALL：急性淋巴细胞白血病；MAL：急性混合细胞白血病；MDS：骨髓增生异常综合征；SAA：重型再生障碍性贫血；SR-aGVHD：糖皮质激素耐药型急性移植物抗宿主病

二、短期疗效

1. 总体疗效：全部64例SR-aGVHD患者治疗后第7、14、28天的有效（CR+PR）率分别为48.4％（31/64）、53.1％（34/64）、79.7％（51/64）。治疗第28天CR率54.7％（35/64），PR率25.0％（16/64），13例（20.3％）患者疗效评估为NR。至随访截止，64例患者中有13例NR，51例治疗有效（CR+PR），中位起效时间为7（2～28）d。详见表2。

**表2:** 64例异基因造血干细胞移植后SR-aGVHD患者重组抗CD25人源化单克隆抗体的近期疗效

疗效指标	例（%）
第7天	
CR	6（9.4）
CR+PR	31（48.4）
第14天	
CR	16（25.0）
CR+PR	34（53.1）
第28天	
CR	35（54.7）
CR+PR	51（79.7）

**注** SR-aGVHD：糖皮质激素耐药型急性移植物抗宿主病；CR：完全缓解；PR：部分缓解

2. 人源化CD25单抗治疗不同受累器官SR-aGVHD的疗效：单一皮肤受累的患者2例，均于人源化CD25单抗治疗第28天内获得CR；包含皮肤累及的多器官受累患者共34例，33例获得CR，1例PR。单一肠道受累患者16例，分别有13、3例在治疗第28天内获得CR、PR；包含肠道累及的多器官受累患者共42例，分别有20、10例肠道aGVHD获得CR、PR，12例获得NR。单一肝脏受累患者1例，获得CR；包含肝脏累及的多器官受累患者共26例，分别有8、6、12例患者肝脏aGVHD获得CR、PR、NR。人源化CD25单抗治疗皮肤受累患者的有效率高于肠道受累患者［100.0％（36/36）对79.3％（46/58），*P*＝0.003］，肠道受累患者的有效率高于肝脏受累患者［79.3％（46/58）对55.6％（15/27），*P*＝0.037］。皮肤、肠道、肝脏受累患者的中位起效时间分别为3（1～10）d、7（2～28）d、6（2～27）d。单器官受累SR-aGVHD患者的有效率明显高于≥2个器官受累患者［100.0％（19/19）对71.1％（32/45），*P*＝0.007］。详见表3。

**表3:** 重组抗CD25人源化单克隆抗体治疗不同器官受累SR-aGVHD患者的疗效［例（％）］

受累器官	例数	第7天有效	第14天有效	第28天有效
单器官受累				
皮肤	2	2（100）	2（100）	2（100）
肠道	16	12（75.0）	13（81.2）	16（100）
肝脏	1	1（100）	1（100）	1（100）
2个器官受累				
皮肤+肠道	19	9（47.4）	10（52.6）	18（94.7）
皮肤+肝脏	3	3（100）	3（100）	3（100）
肠道+肝脏	11	2（18.2）	3（27.3）	5（45.5）
3个器官受累				
皮肤+肠道+肝脏	12	2（16.7）	2（16.7）	6（50.0）

**注** SR-aGVHD：糖皮质激素耐药型急性移植物抗宿主病

3. 影响人源化CD25单抗治疗SR-aGVHD第28天疗效的单因素及多因素分析：单因素分析结果显示，受累器官数目（*P*＝0.008）、aGVHD肝脏受累（*P*＝0.001）、Ⅲ/Ⅳ度aGVHD（*P*＝0.041）、aGVHD发生前合并活动性感染（*P*＝0.014）是影响人源化CD25单抗治疗SR-aGVHD疗效的危险因素。多因素分析结果显示，肝脏受累与疗效不佳密切相关（*OR*＝9.588，95％*CI* 0.004～0.291，*P*＝0.002）。详见表4。

**表4:** 影响SR-aGVHD患者重组抗CD25人源化单克隆抗体治疗第28天疗效的单因素分析［例（%）］

影响因素	例数	有效	*P*值
患者性别			0.755
男	38	31（81.6）	
女	26	20（76.9）	
患者年龄			0.343
≤40岁	42	35（83.3）	
>40岁	22	16（72.7）	
供者性别			0.732
男	47	38（80.9）	
女	17	13（76.5）	
造血干细胞来源			0.757
外周血	34	28（82.4）	
骨髓	30	23（76.7）	
脐血干细胞输注			0.751
有	40	31（77.5）	
无	24	20（83.3）	
供者类型			0.606
同胞全相合	12	10（83.3）	
单倍体	49	38（77.6）	
无关供者	3	3（100.0）	
供患者血型			0.751
不合	24	20（83.3）	
相合	40	31（77.5）	
回输CD34^+^细胞数			0.536
≥中位数	32	24/32（75.0）	
<中位数	31	26/31（83.9）	
回输CD3^+^细胞数			0.982
≥中位数	33	26/33（78.8）	
<中位数	26	21/26（80.8）	
受累器官数			0.008
1个	19	19（100）	
2个	33	28（84.8）	
3个	12	7（58.3）	
肝脏受累			<0.001
有	27	15（55.6）	
无	37	36（97.3）	
肠道受累			0.333
有	58	45（77.6）	
无	6	6（100）	
aGVHD分级			0.041
Ⅱ度	13	13（100）	
Ⅲ/Ⅳ度	51	38（74.5）	
用药前活动性感染			0.014
有	29	19（65.5）	
无	35	32（91.4）	

**注** SR-aGVHD：糖皮质激素耐药型急性移植物抗宿主病。有效例数为完全缓解与部分缓解例数之和

三、影响人源化CD25单抗治疗SR-aGVHD的生存分析

从人源化CD25单抗治疗开始随访，所有患者的中位随访时间为17.1（0.2～50.8）个月。64例SR-aGVHD患者应用人源化CD25单抗治疗后6、12、24个月的OS率分别为69.8％（95％*CI* 64.0％～75.6％）、63.2％（95％*CI* 57.1％～69.3％）、52.6％（95％*CI* 46.1％～59.1％）（图1）。

**图1:**
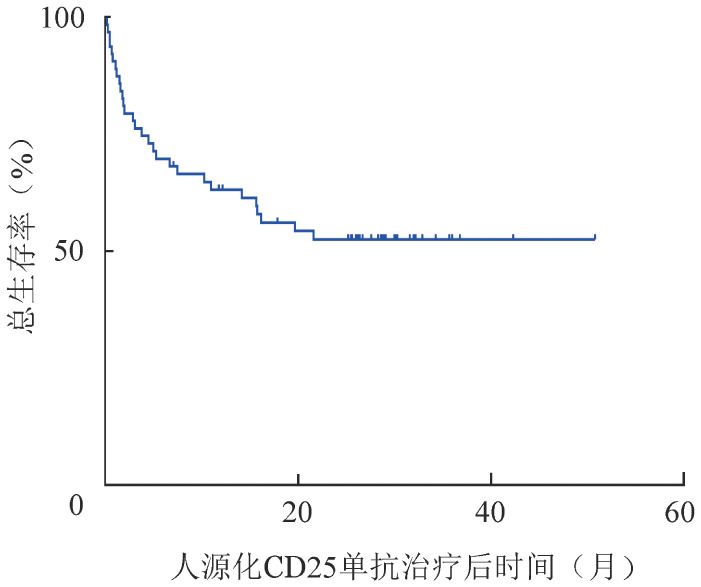
重组抗CD25人源化单克隆抗体治疗64例糖皮质激素耐药型急性移植物抗宿主病患者的总生存曲线

人源化CD25单抗治疗第28天获得CR、PR、NR患者的中位OS时间分别为26.7、15.0、1.2个月，6个月OS率分别为91.4％（95％*CI* 86.7％～96.1％）、79.8％（95％*CI* 69.3％～90.3％）、0（*P*<0.001）（图2）。将患者性别、年龄、移植类型、回输干细胞数量等因素纳入生存分析，结果显示有肝脏累及（*OR*＝0.308，95％*CI* 0.108～0.876，*P*＝0.027）和Ⅲ/Ⅳ度GVHD（*OR*＝9.438，95％*CI* 1.211～73.577，*P*＝0.032）是影响OS的独立危险因素（表5）。

**图2:**
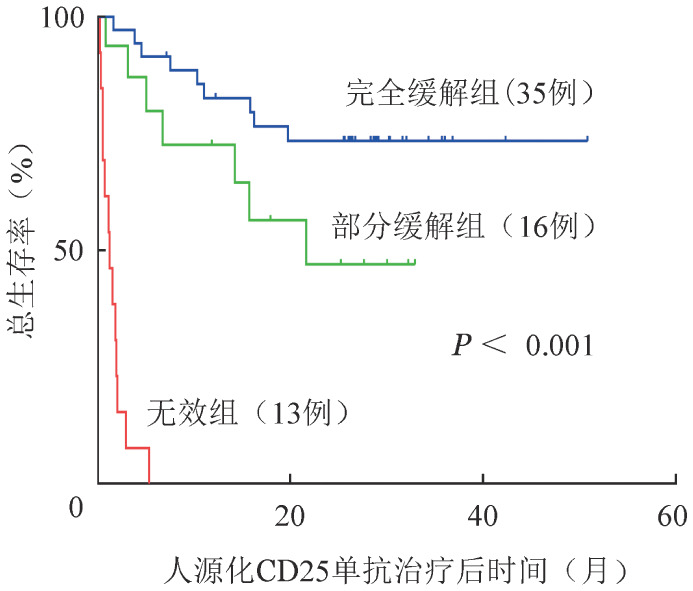
重组抗CD25人源化单克隆抗体治疗28天获得完全缓解（CR）、部分缓解（PR）和无效（NR）患者的总生存曲线

**表5:** 糖皮质激素耐药急性移植物抗宿主病（aGVHD）患者重组抗CD25人源化单克隆抗体治疗后总生存影响因素的分析结果

影响因素	单因素分析		多因素分析	
	*χ^2^*值	*P*值	*P*值	*OR*值（95%*CI*）
患者性别（男，女）	0.317	0.573		
年龄（≤40岁，>40岁）	1.042	0.307		
供者性别（男，女）	2.320	0.128		
造血干细胞来源（外周血，骨髓）	0.539	0.463		
脐血干细胞输注（有，无）	0.003	0.956		
供者类型（同胞全相合，单倍体，无关供者）	2.084	0.353		
供患者血型（血型不合，血型相合）	0.052	0.819		
回输CD34^+^细胞数（≥中位数，<中位数）	0.216	0.642		
回输CD3^+^细胞数（≥中位数，<中位数）	1.094	0.579		
用药前活动性感染（有，无）	7.481	0.006	0.056	0.464(0.211~1.021)
受累器官数（1，2，3）	6.851	0.009	0.587	0.812(0.382~1.723)
肝脏累及（有，无）	12.615	0.000	0.027	0.308(0.108~0.876）
肠道累及（有，无）	3.829	0.050		
GVHD分级（Ⅱ度，Ⅲ/Ⅳ度）	8.436	0.004	0.032	9.438(1.211~73.577)

2. 非复发死亡：64例患者人源化CD25单抗治疗后6、12、24个月NRM率分别为27.0％（95％*CI* 21.4％～32.6％）、28.8％（95％*CI* 23.1％～34.5％）、32.9％（95％*CI* 26.8％～39.0％）。治疗第28天获得CR、PR、NR患者治疗后6个月NRM率分别为5.7％（95％*CI* 1.8％～9.6％）、12.9％（95％*CI* 4.3～21.5％）、100.0％（*P*<0.001）。

3. 生存分析及复发：64例SR-aGVHD患者人源化CD25单抗治疗后6、12、24个月的DFS率分别为66.6％（95％*CI* 60.6％～72.6％）、58.4％（95％*CI* 52.1％～64.7％）、49.8％（95％*CI* 43.4％～56.2％）。治疗第28天获得CR、PR和NR患者的中位DFS时间分别为26.7、12.1、1.2个月。治疗第28天获得CR、PR和NR患者治疗后6个月DFS率分别为88.6％（95％*CI* 83.2％～94.0％）、72.5％（95％*CI* 60.7％～72.5％）、0（*P*<0.001）。64例SR-aGVHD患者中共有11例复发，中位复发时间为移植后8.8（3.2～20.0）个月。造血干细胞移植后6、12个月的累积复发率分别为8.5％（95％*CI* 4.4％～12.6％）、17.7％（95％*CI* 12.0％～23.4％）。

四、感染

64例SR-aGVHD患者人源化CD25单抗治疗后6个月内共42例（65.6％）发生感染并发症，包括巨细胞病毒（CMV）感染25例次（39.1％）、EB病毒感染12例次（18.8％）、出血性膀胱炎21例次（32.8％），17例（26.6％）患者发生其他感染（肺部感染10例次、肠道感染8例次、中枢神经系统感染2例次、软组织感染和败血症各2例次）。

五、患者死亡情况

所有患者的中位随访时间为17.1（0.2～50.8）个月，至随访截止，29例患者死亡，死亡原因包括白血病复发9例、严重感染9例、aGVHD 7例、血栓性微血管病（TMA）2例、其他2例。9例死于严重感染患者的中位死亡时间为人源化CD25单抗治疗后3.1（1.2～15.9）个月。

## 讨论

SR-aGVHD的治疗目前尚无标准方案，CD25单抗是临床使用比较广泛的二线治疗方案之一。目前国内临床可应用的CD25单抗有人源化CD25单抗和鼠/人嵌合型CD25单抗两类，二者结构不完全相同[9]。

人源化CD25单抗daclizumab（达利珠单抗）治疗SR-aGVHD第28天的有效率为29％～68％[10]，鼠/人嵌合型CD25单抗治疗SR-aGVHD第28天的有效率为65％～85％[11]–[14]。Tao等[15]报道人源化CD25单抗治疗SR-aGVHD第28天有效率为83％，CR率为58％。本研究中，人源化CD25单抗治疗第28天的有效率为84.4％，CR率为54.7％，与既往发表研究结果相似。

SR-aGVHD患者预后不佳，2年OS率为25％，2年NRM为65％[16]。目前已发表的研究中，CD25单抗治疗SR-aGVHD的1年OS率为47％～64％[12],[17]–[18]，1年NRM为32.6％[17]。本研究中，人源化CD25单抗治疗SR-aGVHD后12个月OS率、NRM分别为63.2％（95％*CI* 57.1％～69.3％）、32.9％（95％*CI* 26.8％～39.0％），与既往报道相符。

磷酸芦可替尼是目前治疗SR-aGVHD的常用二线药物，Jagasia等[19]报道磷酸芦可替尼治疗SR-aGVHD第28天有效率、CR率分别为54.9％、26.8％，1年OS率、NRM分别为42.6％、52.9％。目前，尚无人源化CD25单抗和芦可替尼治疗SR-aGVHD疗效比较的研究数据。

本研究中，人源化CD25单抗治疗第28天有效（CR+PR）患者的预后明显优于NR患者，这与文献[12],[15]–[17]报道的结果是一致的，提示对人源化CD25单抗治疗有效的患者可以获得生存改善。

以往研究表明，CD25单抗对于aGVHD的治疗效果以皮肤受累最佳，肠道受累次之，肝脏受累最差[11]–[12],[14],[17]。也有学者报道，肠道受累患者疗效优于皮肤受累患者，皮肤受累患者优于肝脏受累患者[15]。本研究中，人源化CD25单抗的疗效以皮肤受累最佳，肠道受累次之，肝脏受累最差，并且单器官受累SR-aGVHD患者的有效率明显优于≥2个器官受累SR-aGVHD患者。

本研究将可能影响SR-aGVHD疗效的相关临床因素进行分析，结果显示，肝脏受累与治疗第28天疗效不佳密切相关，而Ⅲ/Ⅵ度aGVHD和肝脏受累是影响患者OS的独立危险因素，与文献[12],[14],[17],[20]报道的结果一致。

感染是SR-aGVHD患者应用二线治疗的常见并发症。接受鼠/人嵌合型CD25单抗、ATG、MMF及芦可替尼治疗SR-aGVHD患者的感染发生率分别为59.6％～73.6％[11],[20]、67％[21]、23％～67％[22]–[23]、42.0％～80.3％[24]–[25]。本组患者用药后6个月内共42例（65.6％）发生感染，有9例患者因重症感染死亡，虽然其中5例患者死亡时间为用药3个月之后，人源化CD25单抗对这些患者的感染发展影响可能不大，但感染仍然是aGVHD患者死亡的主要原因之一。

本研究结果显示，人源化CD25单抗是治疗SR-aGVHD的有效药物且相对安全。对人源化CD25单抗治疗有效的患者具有较好的预后。而重度aGVHD，尤其是累及肝脏的患者，仍需探索、优化治疗方案。
